# A controlled open clinical trial of the positive effect of a physical intervention on quality of life in schizophrenia

**DOI:** 10.3389/fpsyt.2023.1066541

**Published:** 2023-02-24

**Authors:** Viviane Batista Cristiano, Michele Fonseca Szortyka, Paulo Belmonte-de-Abreu

**Affiliations:** ^1^Graduate Program in Psychiatry and Behavioral Sciences, Federal University of Rio Grande do Sul, Porto Alegre, Rio Grande do Sul, Brazil; ^2^Schizophrenia Program of Hospital de Clínicas de Porto Alegre, Porto Alegre, Rio Grande do Sul, Brazil; ^3^Sports, Physiotherapy and Physical Activity National Society (SONAFE), Porto Alegre, Brazil; ^4^Department of Psychiatry, Schizophrenia Program of the Federal University of Rio Grande do Sul Medical School, Hospital de Clínicas de Porto Alegre, Porto Alegre, Rio Grande do Sul, Brazil

**Keywords:** quality of life, schizophrenia, sedentary lifestyle, aerobic exercise, functional exercise

## Abstract

**Justification:**

Schizophrenia is a severe mental disorder associated with important physical (obesity and low motor functional capacity) and metabolic (diabetes and cardiovascular diseases) changes that contribute to a more sedentary lifestyle and a low quality of life.

**Objective:**

The study aimed to measure the effect of two different protocols of physical exercise [aerobic intervention (AI) versus functional intervention ([FI)] on lifestyle in schizophrenia compared with healthy sedentary subjects.

**Methodology:**

A controlled clinical trial involving patients diagnosed with schizophrenia from two different locations [Hospital de Clinicas de Porto Alegre (HCPA) and Centro de Atenção Psicosocial (CAPS) in the city of Camaquã] was carried out. The patients undertook two different exercise protocols (IA: 5-min warm-up of comfortable intensity; 45 min of aerobic exercise of increasing intensity using any of the three modalities—a stationary bicycle, a treadmill, or an elliptical trainer; and 10 min of global stretching of large muscle groups; and FI: a 5 min warm-up with a stationary walk; 15 min of muscle and joint mobility exercises; 25 min of global muscle resistance exercises; and 15 min of breathing body awareness work) twice a week for 12 weeks and were compared with physically inactive healthy controls. Clinical symptoms (BPRS), life quality (SF-36), and physical activity levels (SIMPAQ) were evaluated. The significance level was *p* ≤ 0.05.

**Results:**

The trial involved 38 individuals, of which 24 from each group performed the AI, and 14 from each group underwent the FI. This division of interventions was not randomized but was instead decided upon for convenience. The cases showed significant improvements in quality of life and lifestyle, but these differences were greater in the healthy controls. Both interventions were very beneficial, with the functional intervention tending to be more effective in the cases and the aerobic intervention more effective in the controls.

**Conclusion:**

Supervised physical activity improved life quality and reduced sedentary lifestyle in adults with schizophrenia.

## Introduction

Severe mental disorders, such as schizophrenia, are associated with various personal impairments, including cognitive, physical, and metabolic changes (1, 2). These, in turn, are associated with other diseases (cardiovascular, obesity, and diabetes) (1–3) that are the consequences of an unhealthy lifestyle, mental illness manifestations, and the side effects of drug treatment.

Furthermore, this population undertakes little physical activity, which contributes to these additional metabolic and cardiovascular pathologies, such as diabetes, acute myocardial infarction, and stroke (1–4). Additionally, the diet of these individuals is a significant factor to be considered as obesity and a sedentary lifestyle will lead to the development of metabolic and cardiovascular diseases, particularly considering that many drugs used to control the disease also contribute to weight gain (5).

All these scenarios of a sedentary lifestyle, obesity, and metabolic and/or cardiovascular diseases directly influence the quality of life of these individuals. Therefore, several studies have evaluated the effect of physical activity on people with schizophrenia and demonstrated that increased physical activity may induce changes in functional capacity, social interaction, and pain tolerance, even when compared to sedentary individuals without mental disorders (6, 7). These studies suggest that regular exercise may positively affect individuals with schizophrenia, especially those who are sedentary and overweight. This may occur because of the convergence of increased cellular mitosis, increased metabolism, increased production of endorphins and neurotrophic factors, and muscle and neuronal plasticity (8–12). With this hypothesis in mind, many recent studies have focused on physical activity in this population. Almost all the physical activities mentioned were related to aerobic exercise because it has well-known oxidative effects and results can be achieved in a relatively short period of time (on average, 8 weeks are sufficient for systemic responses (13). Another format of physical activity that can be very beneficial for this population and that has not yet been studied is functional training as it uses everyday movements, such as sitting and standing, pulling and pushing, and spinning, thus favoring and stimulating the individual’s autonomy.

The main results of the studies focused on cognitive issues, functional capacity, weight, and biomarkers, revealing positive effects in this population. However, most failed to study the impact on lifestyle and compare it with other physical activities, such as anaerobic exercise. This points to the need for additional data comparing the effect of different types of physical exercise on different health outcomes. Therefore, this study aims to measure the effect of two different physical activity protocols, aerobic intervention (AI) versus functional intervention (FI), on lifestyle in individuals with schizophrenia compared with healthy sedentary individuals.

## Materials and methods

### Trial design

In this section, we describe the clinical trial of physical intervention [aerobic physical intervention (AI) and functional physical intervention (FI)] in two groups of stable outpatients with a diagnosis of schizophrenia (SCZ) and one group of healthy sedentary controls. The AI group received regular care at a public health facility [Psychosocial Attention Center (CAPS)]. Patients under continued outpatient care at CAPS-Camaquã in the surrounding cities of Metropolitan Porto Alegre in southern Brazil received AI, and patients under regular care at a university-based hospital [schizophrenia outpatient clinic (Prodesq) of Hospital de Clínicas de Porto Alegre (HCPA)] received FI.

### Participants

**FIGURE 1:**
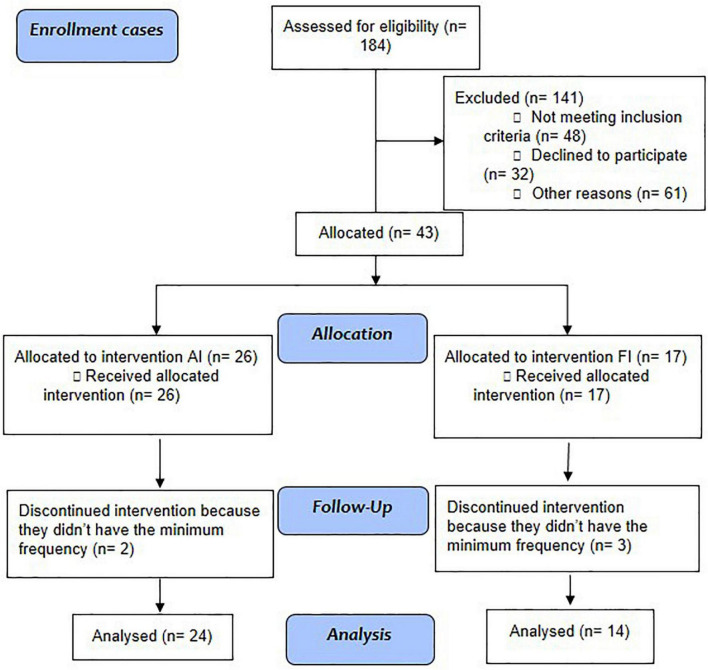
Study flowchart: Aerobic intervention (AI) and functional intervention (FI) in patients with schizophrenia.

Stable outpatients under regular treatment received prior psychiatric diagnosis after a three-step procedure consisting of the following: (a) careful clinical observation with at least three evaluations; (b) a family interview; and (c) a review of their medical records performed by a trained psychiatrist. All met the following inclusion criteria: Diagnostic and Statistical Manual of Mental Disorders, DSM-5; (14) diagnosis of schizophrenia; aged between 18 and 65 years; under stable drug treatment adjusted to their clinical state for at least 3 months; and not involved in any other physical activity programs during the intervention. Patients were recruited from the services where they were being clinically followed (HCPA or CAPES) and were allocated to the intervention group (individuals from HCPA underwent IF, and individuals from CAPES underwent IA) (Figure 1).

The exclusion criteria were as follows: alcohol or other drug abuse in the previous month; major systemic or neurological diseases; physical disability contraindicating physical activity or any physical condition that makes physical activity unsafe; suicide risk confirmed by direct contact with the patient and family; pregnancy or women of reproductive age that did not use a contraception method; and not agreeing to participate in the study after full explanation of the program.

**FIGURE 2:**
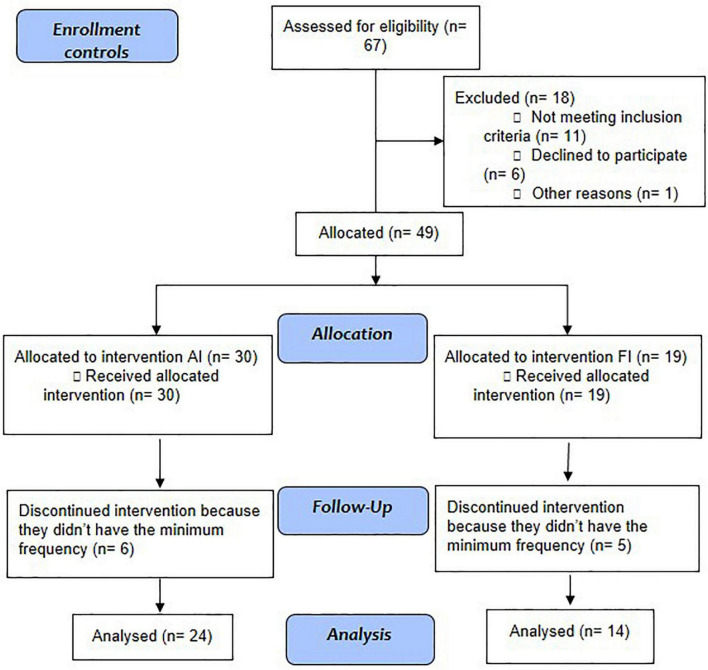
Study flowchart: Aerobic intervention (AI) and functional intervention (FI) in controls.

Controls were recruited through specific social networks (Figure 2). Then, they were paired by sex, age (3 years older or younger), and social class [we followed the classification criteria by classes of the Brazilian Institute of Geography and Statistics (IBGE), which uses the monthly income of all the residents of the same house to list from the richest to the poorest]. Thus, they were divided into the following classes: A (monthly income above R$ 20,900), B (monthly income between R$ 10,450.01 and R$ 20,900), C (monthly income above R$ 4,180 but up to R$ 10,450), D (monthly income between R$ 2,090.01 and R$ 4,180), and E (monthly income of no more than R$ 2,090).

The absence of any major mental illness was defined by a direct interview in which questions about life experiences of memory loss, psychosis (delusions and/or hallucinations), depression, mania, generalized anxiety disorder, and obsessive-compulsive symptoms were asked. Additionally, the subjects were asked about regular physical activity as they were supposed to be sedentary. Exclusion criteria were the same as those applied to patients with SCZ.

### Ethical standards

The authors assert that all procedures contributing to this work complied with the ethical standards of the relevant national and institutional committees on human experimentation and with the Helsinki Declaration of 1975, as revised in 2008.

The study was registered in the Brazilian Research Registry (43408615.7.0000.5327) and the Brazilian Registry of Clinical Trials (ReBEC, ^1^ No. RBR-2h2hjy, registration date 09/29/2020, study start date 07/22/2017) and approved (150066) by the Research Ethics Committee of Hospital de Clínicas de Porto Alegre (HCPA). Patients and their legal guardians provided written informed consent after reading and understanding the intervention program and their rights. Registration took place later because we thought it was not a randomized clinical trial, so there would be no need.

### Clinical assessment

After patient recruitment, previously trained professionals performed a standardized clinical and physical assessment of the study participants before physical intervention and after 3 months of treatment.

### Sedentary lifestyle

The Simple Physical Activity Questionnaire (SIMPAQ) (15) is a 5-item clinical tool designed to assess physical activity among populations at high risk of sedentary behavior. The questionnaire evaluates the last 7 days, including time in bed, sedentary time, time spent walking, type and time spent exercising, and time spent in other activities, including leisure, domestic, work, and transportation activities. It was simultaneously developed and validated in several languages, including English, Spanish, and Portuguese. Following its validation, our group pioneered its use in clinical research.

### Disease severity

The Brief Psychiatric Rating Scale (BPRS) is one of the most used instruments to assess the presence and severity of various psychiatric symptoms and has been in the public domain since 1965 (16). It is valid in several languages, including Portuguese (17), and in Brazil, it is used by the Brazilian Unified Health System (SUS) for monitoring patients. This tool assesses 18 symptom domains, such as somatic worry, anxiety, emotional withdrawal, conceptual disorganization, feelings of guilt, tension, mannerisms and posture, grandiosity, depressed mood, hostility, distrust, hallucinatory behavior, motor retardation, lack of cooperation, unusual thought content, blunted affect, excitement, and disorientation. The assessment takes approximately 5–10 min after an interview with the patient. The clinician then rates each item on a scale ranging from 0 (absent) to 6 (extremely severe) through observation and questioning, depending on the item assessed.

### Quality of life

The Medical Outcomes Study 36-Item Short Form (SF-36) is a commonly used validated questionnaire that is provided in several languages, including Portuguese (18), and has high sensitivity in detecting functional status, among other aspects of quality of life. The SF-36 questionnaire includes eight multiple-item subscales that evaluate functional capacity, physical limitation, pain, general health, vitality, social aspects, emotional limitations, and mental health. The total score on each SF-36 subscale ranges between 0 and 100; the higher the score, the better the patient is.

### Physical intervention

The physical intervention for cases and controls followed an initial assessment that took place after the consent form was read and signed and measured disease severity (BPRS) (cases only), quality of life (SF-36), and physical activity level (SIMPAQ). The aerobic or functional physical intervention program lasted 12 weeks in healthy cases and controls. Patients continued with regular clinical treatment in addition to standardized activity, and after completion of the intervention program, revaluation was performed using all the tests and questionnaires mentioned above.

The aerobic protocol was as follows: 24 patients diagnosed with SCZ were paired with 24 sedentary controls without mental illness. The program lasted 12 weeks and consisted of 1-h aerobic exercise sessions twice a week. The sessions were carried out individually or at most in pairs and monitored by a physiotherapist blinded to the evaluations. The participants were monitored using a Polar FT1^®^ frequency meter with results adjusted for age, sex, weight, and height. Measurements ranged from 70 to 80% of the maximum heart rate calculated using Karvonen’s formula.

A standard aerobic session consisted of the following: a 5-min warm-up at a comfortable intensity followed by aerobic exercise of increasing intensity with one of three modalities: (a) a bicycle ergometer (Embreex 367C, Brazil), (b) a treadmill (Embreex 566BX, Brazil), or (c) an elliptical trainer (Embreex 219, Brazil). This strategy was consistent with public health recommendations that suggest tailoring the program to individual preferences, which has been proven to be feasible in patients diagnosed with SCZ. A trained professional coordinated the intervention sessions with guidance and equipment adjustments and encouraged each participant to perform the exercises in the best way possible. After completing the aerobic exercise, participants globally stretched the major muscle groups.

The functional protocol was as follows: 14 patients diagnosed with SCZ were paired with 14 sedentary controls without mental illness. The program lasted 12 weeks and consisted of 1-h physical function training sessions twice a week. The participants carried out the program in trios or quartets and were trained by a physical therapist blinded to the evaluations.

A standard session consisted of the following: a 5-min warm-up with stationary walking, followed by 15 min of muscle and joint mobility exercises. Then, 25 min of global muscle endurance exercises (paravertebrae, abdominals, extensors, flexors, adductors, hip abductors, flexors and extensors of the shoulders, knees, and elbows) based on the basic movements of functional training (sit and stand, pull and push, and rotate and advance) were performed, followed by 15 min of respiratory body awareness work. A maximum number of repetitions were performed in 30 s (only once per exercise) and accessories such as balls, elastic bands, and dumbbells were used according to the level of resistance required.

### Statistical analyses

The sample size calculation for this study was performed using the WinPepi program (with the evaluation of five co-variables), using a previous study (7) as a baseline. This calculation estimated a minimum number of 30 patients in each group (group 1 patients with SCZ and group 2 controls, for 60 subjects). The normality of the data distribution was assessed using the Kolmogorov–Smirnov test. Quantitative variables with a normal distribution were presented as mean ± standard deviations, while variables with an asymmetric distribution were presented as median and interquartile ranges. Student’s paired *t*-test/independent *t*-test or the Wilcoxon test/Mann–Whitney test were used to compare normal and asymmetric variables, respectively. ANOVA followed by Bonferroni correction was used for comparisons between three or more groups. Relationships between two variables were assessed through Spearman correlation coefficients. Categorical variables were presented as frequencies and analyzed using the Pearson chi-square test, Fisher exact test, or McNemar test. The main outcome measure was assessed using generalized linear model (GLM) analysis (gamma distribution), and confounding factors were determined based on statistical criteria (association with either study factor and the outcome with a *p* ≤ 0.2). The significance level was *p* ≤ 0.05. The analysis of effect size was used to evaluate the magnitude of the difference derived from GLM. SPSS Statistics 22.0 was used to process and analyze data.

## Results

Five of 43 individuals with SCZ who started the exercise protocol were excluded for not having the minimum required frequency (80%, 5 absences over 24 appointments were allowed), whereas in the control group, 49 started, and 11 were excluded for the same reason. Thus, the final sample numbers in both groups was 38 individuals, of which 24 from each group performed the aerobic intervention, and 14 from each group performed the functional intervention. This division of interventions was not randomized but was instead decided upon for convenience. The sociodemographic and clinical data of the samples are shown in Table 1, where we can observe that the groups were homogeneous for sex, weight, and BMI, showing statistical differences only in age and height.

**TABLE 1:** Baseline sample characteristics.

Variables/Group	Cases	Controls	*P*-value
**Gender *n* (%)**			
Male	32 (84.2)	32 (84.2)	–
Female	6 (15.8)	6 (15.8)	
**Schooling *n* (%)**			
Basic education (up to 12 years of schooling)	38 (100)	27 (71.1)	–
Higher education (more than 12 years of schooling)	–	11 (28.9)	–
**Marital status *n* (%)**			
Single	37 (97.4)	11 (28.9)	–
Married	1 (2.6)	27 (71.1)	
**Smoking *n* (%)**			
Yes	14 (36.8)	–	–
No	24 (63.2)	38 (100)	–
**Years of illness *n* (%)**			
<7 years	4 (10.5)	–	–
>7 years	34 (89.5)	–	–
**Antipsychotic medication *n* (%)**			
Typical antipsychotic	13 (34.2)	–	–
Atypical antipsychotic	17 (44.7)	–	–
Combination of typical and atypical	8 (21.1)	–	–
Age (years, mean ± SD)	40.95 ± 11.37	41.68 ± 11.22	0.039*
Weight (kg, mean ± SD)	83.77 ± 23.56	88.66 ± 18.51	0.274
Height (m, mean ± SD)	1.69 ± 0.080	1.73 ± 0.070	0.011*
BMI (mean ± SD)	29.23 ± 7.96	29.55 ± 5.88	0.829
Psychiatric hospitalizations median (p. 25–75)	2.00 (0.75–4.00)	–	–

SD, standard deviation; BMI, body mass index; *p < 0.05. Student’s independent *t*-test for quantitative variables; chi-square test for qualitative variables.

### Brief Psychiatric Rating Scale

Table 2 shows the results of the BPRS clinical scale in the different protocols for both the pre- and post-moments in the case group. There was a worsening of hostility symptoms (*p* = 0.02) in the AI group.

**TABLE 2:** BPRS values in patients according to intervention.

	Total cases (mean ± SD)			
Intervention	Aerobic (*n* = 24)		Functional (*n* = 14)	
Time	Before	After	Before	After
**Variable**				
Anxiety and depression 5-2-9-1	5.71 ± 4.83	5.75 ± 5.11	5.29 ± 5.34	7.36 ± 4.88
Retardation 13-16 3-18-14-7	5.67 ± 6.58	6.38 ± 6.86	5.21 ± 5.18	5.86 ± 4.47
Thinking disorder 11-15-12-4-10-8	6.33 ± 5.30	7.04 ± 5.93	7.36 ± 7.68	8.64 ± 8.19
Activation 7-6-17-8	1.00 ± 2.25	1.96 ± 2.97	1.29 ± 2.61	1.79 ± 2.49
Hostility 10-11-14-8	2.17 ± 2.16	3.83 ± 4.29	2.57 ± 3.74	3.93 ± 4.25

SD, standard deviation; BPRS, Brief Psychiatric Rating Scale. Student’s paired *t*-test.

### 36-Item short form quality of life scale

Patients with SCZ showed an increase of approximately 20% in almost all domains of the SF-36, except for the pain domain, which decreased in the AI group. Even with this clear improvement, only two domains in each intervention (AI, functional capacity, *p* < 0.001, and limitations by emotional aspects, *p* = 0.014; FI, pain, *p* = 0.002, and limitations by emotional aspects, *p* = 0.039) changed significantly (Table 3).

**TABLE 3:** Assessment of quality of life by SF-36 before and after intervention between cases and controls according to intervention.

	Patients (mean ± SD)				Controls (mean ± SD)			
Intervention	Aerobic (*n* = 24)		Functional (*n* = 14)		Aerobic (*n* = 24)		Functional (*n* = 14)	
Time	Before	After	Before	After	Before	After	Before	After
**Variable**								
Functional capacity	64.38 ± 31.98	80.65 ± 20.36	69.29 ± 26.23	73.21 ± 22.41	75.83 ± 16.66	85.25 ± 12.24	78.93 ± 20.86	84.29 ± 17.74
Physical limitations	45.83 ± 45.25	57.61 ± 37.26	39.29 ± 47.75	66.07 ± 38.44	68.83 ± 31.82	79.79 ± 27.99	61.79 ± 29.52	80.36 ± 24.53
Pain	68.79 ± 25.99	61.65 ± 34.43	57.36 ± 32.37	80.36 ± 18.25	59.67 ± 27.15	74.67 ± 23.87	59.57 ± 11.57	80.36 ± 12.48
General health	46.79 ± 23.17	54.82 ± 23.28	55.00 ± 17.72	59.29 ± 21.38	53.42 ± 17.57	61.92 ± 18.39	62.79 ± 14.90	77.14 ± 16.02
Vitality	56.67 ± 26.89	66.14 ± 29.40	56.79 ± 18.57	62.14 ± 26.73	62.08 ± 18.82	71.04 ± 17.51	65.36 ± 15.12	74.64 ± 17.27
Social aspects	56.33 ± 27.00	64.22 ± 24.83	56.79 ± 29.64	69.64 ± 31.28	73.75 ± 28.06	83.21 ± 22.12	74.11 ± 21.63	82.14 ± 21.21
Emotional limitations	38.79 ± 44.66	60.50 ± 44.43	38.09 ± 48.67	66.66 ± 43.39	70.50 ± 30.57	84.33 ± 14.79	73.81 ± 26.76	78.61 ± 30.95
Mental health	64.00 ± 28.48	66.00 ± 26.84	62.86 ± 28.13	68.86 ± 20.29	73.96 ± 20.79	81.63 ± 19.50	72.00 ± 11.09	78.57 ± 17.37

ANOVA test followed by Bonferroni correction.

By contrast, the control group improved significantly in seven of the SF-36 domains in the AI (functional capacity *p* < 0.001; limitations by physical aspects, *p* = 0.013; pain, *p* = 0.001; vitality, *p* = 0.002; social aspects, *p* = 0.0271; limitations by emotional aspects, *p* = 0.014; and mental health, *p* = 0.024) and in four domains in the FI (limitations by physical aspects, *p* = 0.013; pain, *p* < 0.001; general health status, *p* < 0.001; and vitality, *p* = 0.049) (Table 3).

### Simple Physical Activity Questionnaire

The AI case group showed a significant change in exercise time from 16 to 126 min per week (*p* < 0.001) but not in the other SIMPAQ items. The FI cases, on the other hand, also showed significant improvement in exercise time from 0 to 110 min per week (*p* < 0.001) and in weekly walking time (from 108 to 177 min/week; *p* < 0.001) (Table 4).

**TABLE 4:** Simple Physical Activity Questionnaire (SIMPAQ) assessment according to intervention.

	Patients (mean ± SD)				Controls (mean ± SD)			
Intervention	Aerobic (*n* = 24)		Functional (*n* = 14)		Aerobic (*n* = 24)		Functional (*n* = 14)	
Time	Before	After	Before	After	Before	After	Before	After
**Variable**								
Time in bed (min)	607.5 ± 114.07	586.25 ± 84.84	660.36 ± 133.14	615.71 ± 75.72	466.25 ± 82.51	422.92 ± 67.02	437.14 ± 57.27	420.71 ± 73.85
Sedentary time (min)	365.0 ± 144.73	368.75 ± 163.0	268.57 ± 250.60	225.0 ± 179.95	427.71 ± 107.52	337.29 ± 111.79	387.76 ± 236.65	465.0 ± 285.52
Time spent walking (min)	161.25 ± 211.87	195.42 ± 271.50	108.57 ± 206.24	177.14 ± 191.60	132.92 ± 150.52	309.88 ± 309.40	269.29 ± 247.87	338.57 ± 258.57
Type and time spent for exercises (min)	16.67 ± 44.40	126.67 ± 140.76	0	110,0 ± 47.72	20.0 ± 70.03	202.5 ± 175.46	137.86 ± 116.57	207.57 ± 107.42
Time spent for other physical activities (min)	34.58 ± 76.44	42.92 ± 75.84	4.29 ± 16.04	8.57 ± 24.76	69.38 ± 107.42	158.54 ± 180.42	15.00 ± 27.10	38.93 ± 51.60

ANOVA test followed by Bonferroni.

The AI control group showed significant improvement in three measures: (a) sedentary time (from 427 to 337 min/day; *p* = 0.007), (b) walking time (from 132 to 309 min/week; *p* = 0.004), and (c) exercise time (from 20 to 202 min/week; *p* < 0.001). The FI control, on the other hand, had only showed a significant improvement in exercise time (from 137 to 207 min/week; *p* < 0.001) (Table 4).

## Discussion

The results of the study on quality of life and levels of physical inactivity are promising and unprecedented, as the cases were compared with healthy controls and submitted to two different interventions. Of the eight domains of quality of life assessed by the SF-36, only two showed statistically significant differences in the AI cases (functional capacity and limitations by emotional aspects) in the comparison between before and after. Additionally, only two domains were statistically significantly different in the FI cases (pain and limitations by emotional aspects). The healthy AI controls showed significant improvement in seven domains of quality of life (pain, limitations by physical aspects, limitations by emotional aspects, general health status, social aspects, functional capacity, and vitality domain), whereas the FI controls improved in four domains in the before and after comparison (limitations by physical aspects, pain, general health status, and vitality). These results allow us to conclude that the benefits of physical activity, independent of the protocol, were superior in healthy controls, and this should be better investigated in future studies. Could the disease be responsible for this difference in response?

The sedentary lifestyle levels assessed by SIMPAQ were different from those of quality of life, with the healthy control group AI and the case group FI showing more significant results in the before and after comparison. This demonstrates that the groups respond better to a specific type of physical exercise, even though they are homogeneous and have the same previous levels of physical inactivity.

Most of the studies available in the literature with this population (SCZ) proposed an aerobic-type activity and their results, in the great majority, were positive in the symptoms of the disease, however our findings were opposite to them, highlighting the hostility variable of the BPRS, where individuals who performed aerobic exercise showed a significant worsening of symptoms (9–11). What we can safely say is that both interventions produced positive results in cases and in controls in terms of quality of life, favoring the change in the lifestyle of these individuals, taking them out of the “comfort zone” of their sedentary lifestyle, which had made them inactive, and leading them to a more healthy life. After all, physical inactivity is responsible for numerous pathologies, such as obesity and cardiovascular diseases, which are both very prevalent with SCZ. Data from the WHO indicate that the world population is experiencing a sedentary lifestyle; therefore, any physical activity should be encouraged and should last at least 75–150 min per week (19).

When we look at each variable individually, we can see that the quality of life pain domain improved scores in cases and in controls. However, although this difference occurred for both the AI and FI in the controls, it only occurred for the FI in the cases. The same occurred for the variable time spent in other activities of SIMPAQ. These two differences suggest that the functional protocol tends to produce better responses in SCZ patients. Few studies (20, 21) have evaluated this in terms of functional and/or postural activities in this population; therefore, it is necessary to explore new lines of research with this type of physical activity. Thus, we will feel more confident when creating protocols. Furthermore, our results emphasize the correlation between the quality of life and the physical activity levels in this population, a hypothesis that has already been discussed previously with psychoses (22). Another critical variable to be considered in studies on physical activities in this population is sleep, as recent studies have shown its correlation with sports performance, the development of chronic and inflammatory diseases (diabetes and cardiovascular diseases), and mental disorders (depression and psychoses) (23, 24).

## Limitations

Our study has significant limitations, such as the small sample size. Additionally, our sample was mostly male and was already in the chronic course of the disease. Some of these factors occurred due to a selection bias, in which there was a higher male prevalence in the centers of origin (CAPES and HCPA) of individuals SCZ; to avoid interference in the results, statistical adjustments were made. Furthermore, because we depended on a specific structure and specific equipment to carry out the physical exercise protocols, we divided the groups by convenience and not by randomization. It would have been interesting to divide the group of individuals with SCZ into sedentary and non-sedentary groups for a better comparison with healthy controls. Still, we were again restricted to the profile of the patients in the centers studied, where the majority were sedentary. We suggest further research should include a nutritional control associated with physical activity, as well as additional clinical questionnaires about the disease.

## Conclusion

The results of our study are significant and unprecedented for this population. We were pioneers in comparing different physical protocols and assessing lifestyle, as well as the importance of quality of life. Our findings, although preliminary, prove the effectiveness of the practice of guided physical activity among patients with a severe mental disorder, such as SCZ, and serve to guide further studies.

## Data availability statement

The raw data supporting the conclusions of this article will be made available by the authors, without undue reservation.

## Ethics statement

The studies involving human participants were reviewed and approved by Research Ethics Committee of Hospital de Clínicas de Porto Alegre (HCPA). The patients/participants provided their written informed consent to participate in this study.

## Author contributions

All authors listed have made a substantial, direct, and intellectual contribution to the work, and approved it for publication.

## Footnotes

1
https://ensaiosclinicos.gov.br/rg/RBR-2h2hjy

## References

[1] Vancampfort D Correll C Galling B Probst M De Hert M Ward P Diabetes mellitus in people with schizophrenia, bipolar disorder and major depressive disorder: a systematic review and large scale meta-analysis. *World Psychiatry.* (2016) 15:166–74. 10.1002/wps.20309 27265707 PMC4911762

[2] Moreno-Küstner B Martín C Pastor L. Prevalence of psychotic disorders and its association with methodological issues: a systematic review and meta-analyses. *PLoS One.* (2018) 13:e0195687. 10.1371/journal.pone.0195687 29649252 PMC5896987

[3] Nemani K Li C Olfson M Blessing E Razavian N Chen J Association of psychiatric disorders with mortality among patients with covid-19. *JAMA Psychiatry.* (2021) 78:380–6. 10.1001/jamapsychiatry.2020.4442 33502436 PMC7841576

[4] Soares D Carvalho D Ribeiro M Diniz E Rêgo AN. Prevalence and predictors of treatment-resistant schizophrenia in a tertiary hospital in Northeast Brazil. *Trends Psychiatry Psychother.* (2021) 43:270–7. 10.47626/2237-6089-2020-0151 34139114 PMC8835382

[5] Alvarez-Herrera S Escamilla R Medina-Contreras O Saracco R Flores Y Hurtado-Alvarado G Immunoendocrine peripheral effects induced by atypical antipsychotics. *Front Endocrinol (Lausanne).* (2020) 21:195. 10.3389/fendo.2020.00195 32373066 PMC7186385

[6] Vancampfort D Stubbs B Mitchell A De Hert M Wampers M Ward P Risk of metabolic syndrome and its components in people with schizophrenia and related psychotic disorders, bipolar disorder and major depressive disorder: a systematic review and meta-analysis. *World Psychiatry.* (2015) 14:339–47. 10.1002/wps.20252 26407790 PMC4592657

[7] Cristiano V Vieira Szortyka M Lobato M Ceresér K Belmonte-de-Abreu P. Postural changes in different stages of schizophrenia is associated with inflammation and pain: a cross-sectional observational study. *Int J Psychiatry Clin Pract.* (2016) 21:104–11. 10.1080/13651501.2016.1249892 27868463

[8] Vancampfort D Probst M Sweers K Maurissen K Knapen J De Hert M. Relationships between obesity, functional exercise capacity, physical activity participation and physical self-perception in people with schizophrenia. *Acta Psychiatr Scand.* (2011) 123:423–30. 21219266 10.1111/j.1600-0447.2010.01666.x

[9] Firth J Stubbs B Vancampfort D Schuch F Lagopoulos J Rosenbaum S Effect of aerobic exercise on hippocampal volume in humans: a systematic review and meta-analysis. *Neuroimage.* (2018) 166:230–8. 10.1016/j.neuroimage.2017.11.007 29113943

[10] Abdul Rashid N Nurjono M Lee J. Clinical determinants of physical activity and sedentary behavior in individuals with schizophrenia. *Asian J Psychiatr.* (2019) 46:62–7. 10.1016/j.ajp.2019.10.004 31627166

[11] Ospina L Wall M Jarskog L Ballon J McEvoy J Bartels M Improving cognition via exercise (ICE): study protocol for a multi-site, parallel-group, single-blind, randomized clinical trial examining the efficacy of aerobic exercise to improve neurocognition, daily functioning, and biomarkers of cognitive change in individuals with schizophrenia. *J Psychiatr Brain Sci.* (2019) 4:e190020. 10.20900/jpbs.20190020 31938726 PMC6958554

[12] De Sousa R Improta-Caria A Aras-Júnior R de Oliveira E Soci Ú Cassilhas R. Physical exercise effects on the brain during COVID-19 pandemic: links between mental and cardiovascular health. *Neurol Sci.* (2021) 42:1325–34. 10.1007/s10072-021-05082-9 33492565 PMC7829117

[13] Neufer P. The effect of detraining and reduced training on the physiological adaptations to aerobic exercise training. *Sports Med.* (1989) 8:302–20. 10.2165/00007256-198908050-00004 2692122

[14] American Psychiatric Association [APA]. *Diagnostic and Statistical Manual of Mental Disorders, (DSM-5).* 5th ed. Arlington, VA: American Psychiatric Association (2013).

[15] Rosenbaum S Morell R Abdel-Baki A Ahmadpanah M Anilkumar T Baie L Assessing physical activity in people with mental illness: 23-country reliability and validity of the simple physical activity questionnaire (SIMPAQ). *BMC Psychiatry.* (2020) 20:108. 10.1186/s12888-020-2473-0 32143714 PMC7060599

[16] Overall J Gorham D. The brief psychiatric rating scale. *Psychol Rep.* (1962) 10:799–812.

[17] Crippa J Sanches R Hallak J Loureiro S Zuardi A. Factor structure of Bech’s version of the Brief psychiatric rating scale in Brazilian patients. *Braz J Med Biol Res.* (2002) 35:1209–13. 10.1590/s0100-879x2002001000014 12424494

[18] Ciconelli R Ferraz M Santos W Meinão I Quaresma M. Tradução para a língua portuguesa e validação do questionário genérico de avaliação de qualidade de vida SF-36 (Brasil SF-36)/Brazilian-Portuguese version of the SF-36. A reliable and valid quality of life outcome measure. *Rev Bras Reumatol.* (1999) 39:143–50.

[19] World Health Organization [WHO]. *Physical Activity.* Geneva: World Health Organization (2020).

[20] Akbas E Erdem E Gunes Yalcin E Özkan T Kinikli G. Effects of pilates-based exercises on functional capacity and mental health in individuals with schizophrenia: a pilot study. *Physiother Theory Pract.* (2021) 24:1–9. 10.1080/09593985.2021.1929613 34030579

[21] Tréhout M Leroux E Bigot L Jego S Leconte P Reboursière E A web-based adapted physical activity program (e-APA) versus health education program (e-HE) in patients with schizophrenia and healthy volunteers: study protocol for a randomized controlled trial (PEPSY V@Si). *Eur Arch Psychiatry Clin Neurosci.* (2021) 271:325–37. 10.1007/s00406-020-01140-z 32458107

[22] Gouveia R Ferreira-Junior A Schuch F Zanetti G da Silva A Del-Bem C Physical activity, quality of life and global functioning an early stages of psychosis. *Psychiatr Danub.* (2020) 32:373–9. 10.24869/psyd.2020.373 33370735

[23] Adams M Katz D Shenson D. A healthy lifestyle composite measure: significance and potential uses. *Prev Med.* (2016) 84:41–7. 10.1016/j.ypmed.2015.12.005 26724520

[24] Werneck A Vancampfort D Stubbs B Silva D Cucato G Christofaro D Prospective associations between multiple lifestyle behaviors and depressive symptoms. *J Affect Disord.* (2022) 301:233–9. 10.1016/j.jad.2021.12.131 34986379

